# Microbiology of rhinosinusitis in immunosupressed patients from the University Hospital

**DOI:** 10.1590/S1808-86942011000400018

**Published:** 2015-10-19

**Authors:** Erica Ortiz, Ronny Tah Yen Ng, Fernando Canola Alliegro, Cristiane Teixeira, Eder Barbosa Muranaka, Eulalia Sakano

**Affiliations:** 1 Medical specialist in otorhinolaryngology, master's degree and doctoral degree in otorhinolaryngology, Medical Science School (Ciências Médicas - FCM), UNICAMP. Collaborating otorhinolaryngologist at the Rhinology Unit, Otorhinolaryngology and Head & Neck Surgery Discipline, UNICAMP. Graduate course at the FCM-UNICAMP; 2 Graduate student, Otorhinolaryngology Discipline, FCM-UNICAMP. Medical otorhinolaryngologist; 3 Resident, Otorhinolaryngology and Head & Neck Surgery Discipline, FCM-UNICAMP; 4 Medical otorhinolaryngologist and past resident of the Otorhinolaryngology Discipline, UNICAMP; 5 Resident, Otorhinolaryngology and Head & Neck Surgery Discipline, UNICAMP; 6 Assistant professor, Otorhinolaryngology and Head & Neck Surgery Discipline, UNICAMP. Faculty member, Otorhinolaryngology and Head & Neck Surgery Discipline, UNICAMP

**Keywords:** immunosuppression, microbiology, sinusitis

## Abstract

**Abstract:**

Immunosuppressed patients are often susceptible to upper airway infections, especially those of the paranasal sinuses. These can sometimes jeopardize treatment success and even lead to a fatal outcome.

**Objective:**

To study the paranasal microbiology of immunosuppressed patients with clinical evidence of rhinosinusitis, and compare it with that from immunocompetent patients.

**Material and method:**

Retrospective study, in which 42 immunosuppressed and 16 immunocompetent patients were selected. All had clinically evident acute or recurrent rhinosinusitis and were submitted to ethmoidal or sphenoid sinusectomy or maxillary sinus puncture to gather material for microbiological cultures.

**Results:**

There were 92% positive cultures, and 21% were negative. Of the positive cultures, 38% were bacterial, with P. aeruginosa being the most frequent agent; 64% were fungal, which occurred in the most immunocompromised patients. In the immunocompetent group, there were 62.5% positive cultures and 37.5% negative ones. All the positive ones were bacterial, with no fungi.

**Conclusion:**

Transplant recipients were prone to develop bacterial rhinosinusitis by Gram positive and Gram negative agents, the most common of the latter being Pseudomonas aeruginosa. Fungal infections occurred in the severely immunosuppressed, and it was absent in immunocompetent patients.

## INTRODUCTION

At present there are more immunosuppressed patients because of medical advances in the treatment of malignancies, degenerative diseases, and autoimmune conditions in patients receiving transplants, radiotherapy, and chemotherapy. According to the Brazilian Ministry of Health, this country has one of the largest organ transplant systems in the world; it is third, after Spain and the United States. There were 23,593 organ, tissue, and cell transplants from January to June 201^0^^1^.

Transplant patients undergo several preparatory organic changes and remain immunosuppressed for variable periods. Immunosuppressant drugs, chemotherapy, radiotherapy, prolonged antibiotic use, graft versus host disease, and long hospital stays increase the susceptibility of these patients to infection, especially upper airways infections. These infections may delay or abort the success of transplants, and may even lead to death. Fungal infections have been reported as the main cause of morbidity and mortality in these patients; this is generally due to severe immunosuppression, delays in accurate diagnoses, uncommon etiological agents, aggressive clinical and surgical therapy, and especially the invasive nature of these infections^2^, ^3^, ^4^, ^5^.

Rhinosinusitis often develops in these patients. Radiographs of the facial sinuses are done routinely when patients present fever; often maxillary sinus opacification is seen, which suggests acute rhinosinusitis. A clinical evaluation of these patients is not always easy, and several of them progress with complicated or recurrent rhinosinusitis because of the abovementioned special features of immunosuppressed patients. It is important to isolate the infectious microorganism for appropriate medical or surgical therapy to be given^2,^^5^.

The main purpose of this study was to investigate the paranasal sinus microbiology in immunosuppressed patients that develop rhinosinusitis, seen at our institution. A second purpose was to compare the results with bacterioscopies of acute rhinosinusitis in immunocompetent patients that were exposed to the same hospital flora, and with known data in the literature.

## MATERIAL AND METHODS

A retrospective descriptive study was carried out in the Otorhinolaryngology Outpatient Unit of the Clinic Hospital. The institutional ethics review board approved the study (protocol 088-2002). There were 42 immunosuppressed adult patients and 16 immunocompetent adult patients, all with a clinical diagnosis of acute rhinosinusitis (fever, headache, rhinorrhea, nasal block, secretory cough). The diagnosis was confirmed by nasal endoscopy in all patients; it revealed rhinorrhea with a discharge (pus or citrine color) in the middle meatus and/or sphenoethmoid recess. Material was collected for cultures and antibiograms by maxillary sinus puncture and/or sphenoid and/or ethmoid sinusectomy. More than one sample was taken in patients with bilateral disease. Gram positive and negative bacteria, micobacteria, and fungi were investigated in MacConkey, blood Agar, and Sabourand cultures. Anaerobic bacteria were not studied because of technical difficulties in the hospital laboratory.

Immunosuppression among the 42 patients was due to hematologic, renal, and hepatic diseases that required transplantation of these organs. These patients were in the post-transplant period, and were classified as secondary immunosuppressed patients according to the transplant protocol for each of these specialties. Immunocompetent patients had normal immunologic status and no secondary immunosuppressive diseases (diabetes mellitus, AIDS). All patients were randomly distributed male and female adults.

The most prevalent bacteria and fungi were verified by frequency distribution analysis, which was related to several immunosuppressive diseases and immunocompetent subjects.

## RESULTS

Table 1 presents the immunosuppressive diseases. Of 42 immunosuppressed patients, 20 had invasive fungal rhinosinusitis, 6 had recurrent rhinosinusitis, and 16 had acute rhinosinusitis. There were two cases of sphenoid rhinosinusitis, one frontal rhinosinusitis, seven cases of ethmoid rhinosinusitis, and 23 cases of maxillary rhinosinusitis. Invasive fungal rhinosinusitis with nasal turbinate and septal involvement was present in the remaining 9 patients.

**Table 1:** Immunosuppressive diseases x number of patients: acute myeloid leukemia (AML), acute lymphoid leukemia (ALL), chronic myeloid leukemia (CML), Hodgkin's lymphoma (HL), multiple myeloma (MM), multiple sclerosis (MS), renal failure (renal), AIDS (acquired immunodeficiency syndrome), AA (aplastic anemia); Post-HSCT (post hematopoietic stem cell transplant).

Primary diagnosis	*n*
CML	14
MM	1
HL	1
ALL	6
AML	6
MS	1
Renal	2
AIDS	1
AA	1
Post-HSCT	9
Total	42

There was one frontal sinusitis, 2 ethmoid cases of sinusitis, and 13 cases of maxillary sinusitis in the immunocompetent patients.

There were 39 positive punctures (92%) - with growth of microorganisms in cultures - and 9 negative punctures (21%) in immunosuppressed patients, as seen on Table 2. There were gram positive and negative bacteria (38%), and fungi (64%), as shown on Table 3.

**Table 2:** Results of cultures of paranasal sinus secretions collected by puncture.

	Culture of secretions (puncture)	
Number of punctures	*Positive*	*Negative*
Immunosuppressed (n = 42)	39 (92%)	9 (21%)
Immunocompetent (n = 21)	15 (62.5%)	6 (37.5%)

**Table 3:** Etiology in punctures with positive cultures.

*Type of patient and number of positive cultures (puncture)*	Etiologic agent in positive punctures	
	*Bacteria*	*Fungi*
Immunosuppressed (n = 39)	15 (38%)	24 (64%)
Immunocompetent (n = 15)	15 (100%)	0

There were 15 positive punctures (62.5%) in immunocompetent patients, and six negative punctures (37.5%), as shown on Table 2. All positive cultures contained bacteria (100%); there were no fungi, as seen on Table 3.

Table 4 shows the microorganisms that were found. *Pseudomonas aeruginosa* was the most frequent bacteria in immunosuppressed and immunocompetent patients. Uncommon bacteria in acute rhinosinusitis among immunocompetent patients were found in immunosuppressed patients (*Xanthomonas maltophilia, Burkholderia cepacia*).

**Table 4:** Microbiology of sinus secretions.

	Result of punctures	
*Microorganisms*	*Immunosuppressed (n=42)*	*Immunocompetent (n=21)*
*Pseudomonas aeruginosa*	5	4
*Staphilococcus aureus*	2	3
*Streptococcus milleri grupo F*	2	0
*Streptococcus pneumoniae*	2	0
*Xanthomonas maltophilia*	1	0
*Burkholderia cepacia*	1	0
*Haemophilus influenzae*	1	1
*Micrococcus sp*	1	0
*Fusarium*	8	0
*Aspergillus sp*	16	0
*Streptococcus sp*	0	1
*Streptococcus epidermidis*	0	1
*Enterobacter cloacae*	0	1
*Enterobacter aurogenes*	0	1
*Serratia marascences*	0	1
*Citrobacter kosea*	0	1
Negative	9	6

Fungi developed in severely immunocompromised patients, hematopoietic stem-cell immediate post-transplant cases, immediate postoperative renal transplant patients, or cases of acute lymphoid leukemia, acute myeloid leukemia, or aplastic anemia.

## DISCUSSION

Rhinosinusitis in immunosuppressed patients may be fatal, or may delay the recovery of these patients. Investigation by specialists and an accurate diagnosis are essential for successful therapy of immunosuppressed patients. It is essential to find the site of infection, because it is the main complication in these patients. Current studies have shown that hematopoietic stem cell transplant recipients are among the most susceptible individuals to nasosinusal infections (37%), similar to heart transplant patients. Respiratory infection occurs in only 4% of renal transplant patients. The incidence of rhinosinusitis in immunocompetent patients is 23%. Often patients are asymptomatic or present mild and non-specific symptoms, except for fever. A prompt and accurate diagnosis may reduce mortality in these patients^1,^^2,^^3,^^5^.

Nasal endoscopy is the gold standard among diagnostic tests; it can show three-dimensional aspects, the color of the mucosa, and the type and location of discharges from the drainage ostia of paranasal sinuses^6^. Nasosinusal secretions may be citrine or purulent in immunosuppressed patients, depending on the degree of immunosuppression. Nasal endoscopy is more important than computed tomography of the paranasal sinuses, as the latter evaluates only the anatomical involvement. Neves et al. have shown that about 40% of computed tomographies of paranasal sinuses in immunosuppressed patients at an intensive care unit were false positives - in these cases, secretion cultures were negative for microorganisms^7^. Para- nasal sinus puncture under local anesthesia - especially of the maxillary sinus - is a straightforward and well-tolerated procedure, and may guide the treatment of rhinosinusitis in immunosuppressed patients. The infecting microorganism can be cultured and identified by this approach. Additionally, the nasosinusal drainage ostium may be seen directly in endoscopy for puncture, the status of the sinus mucosa can be checked, and the cause of obstruction of the ostium may be seen. A few authors have argued that maxillary sinus puncture may be an adjuvant therapy in rhinosinusitis, as it may be used to rinse the sinus with saline and antibiotics. We carry out sinus puncture at our institution to identify the causative agent and to guide antibiotic therapy.

The literature contains papers describing the presence of unusual microorganisms in immunosuppressed patients, especially in leukopenic patients, which may present a fulminating fungal form of rhinosinusitis^2^, ^3^, ^4^, ^5^. This situation is more likely in the immediate transplant postoperative period or when patients are deeply immunosuppressed. This study encountered fungi (A*spergillus fumigattos, Fusarium sp*) in a little over half of the patients with invasive fungal rhinosinusitis. These numbers differ from those in the literature and in our own institution; the reported incidence of fungal rhinosinusitis in immunosuppressed patients has been about 1%. Contrary to common thought, most of the paranasal sinus infections are bacterial (37%), although the microbiology of transplant patients does not follow expected patterns in acute or recurrent rhinosinusitis that are seen in immunocompetent individuals. Our retrospective results include the most severe cases, which may explain the higher incidence of fungal infections. In centers such as the Clinic Hospital, São Paulo University, *P. aeruginosa* has been reported as the most frequent bacteria, although other uncommon bacteria in acute rhinosinusitis, such as *E. coli, E. cloacae, S. maecescens, P. stutzeri* and *Alcaligenes* were also found. Fungal infections included *Penicillium sp, Candida sp, Scedosporium*, and *Aspergillus*^8^. Thus, wide spectrum antibiotics for gram positive and negative bacteria of several species should be used in transplant patients. Although fungi are less frequent, they should be sought in immunosuppressed patients. Fungi are not common in immunocompetent patients.

In the present study, cultures were negative in 9 immunosuppressed patients, possibly because of prior wide spectrum antibiotic use for more than four days before the sinus puncture. Immunosuppressed patients that present fever at our unit are managed according to a protocol that includes a blood culture and the use of fourth generation cephalosporins. Following these procedures, patients are referred to otorhinolaryngologists for an expert evaluation. Negative cultures of paranasal maxillary sinus punctures (13) have also been reported by Iamamura et al. (1999).^8^

Fungal infections in our patients coincided with severe leucopenia, a predisposing factor that has been reported in the literature. The paranasal sinuses of these patients are usually not involved during the initial stages of the infection - computed tomography of the paranasal sinuses are unaltered. Thus, infection may be suspected if there is fever and severe leucopenia (neutrophils <500/ dl) and a yellowish and/or blackish color of the mucosa of the middle turbinates and/or anterior septum and/or middle meatus, as seen on nasal endoscopy^2,^^6^. In these cases, fungi should be sought in samples of the nasal cavity mucosa, as well as in secretions of the affected sinus (if available); cultures and pathology should be carried out as soon as possible to find fungi, which should be confirmed by cultures within the ensuing 3 weeks^2,^^3,^^5,^^6^.

## CONCLUSION

Immunosuppressed patients may acquire bacterial or fungal rhinosinusitis, while immunocompetent patients have only bacterial rhinosinusitis. Bacteria may be gram positive or negative of several species; at our institution, *Pseudomonas aeruginosa* was the most common bacterium.

Fungi were the most common agent in immunosuppressed patients, and may infect more severely immunosuppressed individual; additional care and clinical suspicion is required in the diagnosis, and treatment should be started promptly in these cases.

## References

[1] Ministério da Saude do Brasil. Transplantes. Disponível em http://portal.saude.gov.br. Acessado em agosto de 2010.

[2] Berlinger NT. (1985). Sinusitis in immunodeficient and immunossupressed patients.. Laryngoscope..

[3] Shibuya TY, Momin F, Abella E, Jacobs JR, Karanes C, Ratanatharathorn V (1995). Sinus Disease in the Bone Marrow Transplant population: incidence, risk factors and complications.. Otolaryngol Head Neck Surg..

[4] Thompson AM, Couch M, Zahurak ML, Johnson C, Vogelsang GB. (2002). Risk Factors for post-stem cell transplant sinusitis.. Bone Marrow Transplant..

[5] Savage DG, Taylor P, Blackwell J, Chen F, Szydlo RM, Rule AS, Spencer A (1997). Paranasal Sinusitis following allogenic bone marrow transplant.. Bone Marrow Transplant..

[6] Diretrizes Brasileiras de Rinossinusites. Braz J Otorhinolaryngol.2008; mar, 74(2), supl.

[7] Neves MC, Voegels RL. (2006).

[8] Iamamura R, Voegels R, Sperandio F, Sennes LU, Silva R, Butugan O (1999). Microbiology of sinusitis in patients undergoing bone marrow transplantation.. Otolaryngol Head Neck Surg..

